# Family-Based Interventions for the Prevention of Substance Abuse and Other Impulse Control Disorders in Girls

**DOI:** 10.1155/2014/308789

**Published:** 2014-03-03

**Authors:** K. L. Kumpfer

**Affiliations:** Department of Health Promotion and Education, University of Utah, Salt Lake City, UT 84112, USA

## Abstract

Standardized family-based interventions are the most effective way of preventing or treating adolescent substance abuse and delinquency. This paper first reviews the incidence of adolescent substance abuse worldwide emphasizing gender and causes by etiological risk and protective factors. New epigenetic research is included suggesting that nurturing parenting significantly prevents the phenotypic expression of inherited genetic diseases including substance abuse. Evidence-based family interventions are reviewed including family change theories behind their success, principles and types of family-based interventions, research results, cultural adaptation steps for ethnic and international translation, and dissemination issues. The author's *Strengthening Family Program* is used as an example of how these principles of effective prevention and cultural adaptation can result in highly effective prevention programs not only for substance abuse, but for other impulse control disorders as well. The conclusions include recommendations for more use of computer technologies to cut the high cost of family interventions relative to youth-only prevention programs and increase the public health impact of evidence-based prevention programs. The paper recommends that to reduce health care costs these family-based approaches should be applied to the prevention and treatment of other impulse control disorders such as obesity and type 2 diabetes, sexually transmitted diseases, and delinquency.

## 1. Introduction

Adolescent behavioural health problems are on the rise worldwide particularly for impulse control disorders (ICDs) including substance abuse, delinquency, obesity, delinquency, and HIV/STDs. Contributing to these developmental issues are children growing up in homes of substance abusing, depressed, highly stressed, and dysfunctional parents. Unfortunately, even functional parents are spending less time with their children because of the worldwide economic crisis that has parents working more hours. Even simple things such as the diminishing number of family meals eaten together have been found to have a negative impact on later adolescent's risk for substance use and negative youth development [1]. When parents and extended family members cannot spend much time parenting their children, they have to get more efficient with the little parenting time they have available if they want their children to grow up to be healthy and productive adults. Hence, disseminating effective parenting and family skills training practices more widely could buffer against the current upswing in adolescent problem behaviors, child maltreatment, and health care costs [2, 3].

Family-based prevention programs focus primarily on education and skills training to enhance positive outcomes in youth by reducing salient risk factors and improving protective factors and resilience [4]. The goal of family-based prevention programs is to promote positive youth development by instilling proper parenting and family relational skills and reinforcing behaviors that increase parent/child attachment or love, effective monitoring and discipline skills, and effective communication. These three family protective factors have been found in tested theoretical models using structural equations modeling (SEM) to be the most critical family process mediators of adolescent outcomes for a wide range of positive or negative youth outcomes (i.e., substance use, delinquency, school failure, and teen pregnancy) [5–8].

There is strong evidence that family-based skills training programs result in positive outcomes with participants [9]. Yet, it is important to understand the current social and family climate that is causing the need for this programming to be implemented widely worldwide. Certain elements of family life have been found to be major risk factors for mental health and substance abuse problems. These include lack of bonding with a parent or significant adult, chaotic home environment, ineffective parenting, other family members abusing substances, social isolation, and inconsistent discipline or expression of values [10]. Another critical factor in developing non-Western or developing rural countries found in SEM modeling of etiological mediators is differential generational acculturation that leads to increased family conflict and lack of family bonding and attachment [11].

Family interventions have been shown to be the most effective prevention and treatment interventions for adolescent substance abuse and other negative developmental outcomes in efficacy studies with at least two years of longitudinal outcomes [12, 13]. They are also cost beneficial [14], because the family members learn and practice new skills to improve their interactions to have long-term sustainable impact on positive youth developmental outcomes. These prevention interventions positively benefit not just the one enrolled child or adolescent, but the whole family—parents, siblings, extended family members living at home, and also caretakers such as foster parents. Also, while they are implemented to improve generally one specific problem behavior such as substance abuse, they impact a broad range of other adolescent and adult outcomes such as improved school and job performance, mental health, delinquency, health, and goal attainment. This is one of their major advantages over youth-only school or community-targeted prevention programs for substance abuse. Family interventions can be implemented in a wide variety of settings from universal prevention applications including family home use DVDs or computer programs and in schools, faith communities, and community programs to indicated prevention including in-home case management or clinical treatment.

*Contents of This Review Article.* This paper is divided into four parts. First it reviews the incidence and prevalence of substance abuse worldwide with an emphasis on the increasing use of alcohol and drugs in girls and women. The second part reviews the etiology of problem behaviors in children including tested etiological or theoretical models because prevention interventions must be designed to reduce precursors of the problem to be prevented. Included in this section is research on the gene/environment interaction studies found in new epigenetics studies with mice and children.

The third section covers theories underlying family interventions, the major types of family intervention approaches, core programmatic ingredients of family-based skills training, cost/benefits, and several of the most effective family-based drug prevention programs including multicomponent family interventions combined with other prevention approaches in alternative settings using schools, community family and youth services, behavioral health centers, faith communities, and even the use of media and the Internet to get larger public health impacts. Research suggests improved results from combining evidence-based family and/or youth only programs using different platforms of delivery and engaging multiple service providers that can focus on a broader range of mediators for different problem behaviors.

The author's *Strengthening Families Program* (SFP) will be reviewed in detail including the theoretical basis, program design, core intervention strategies to address postulated mediators, and the evaluation findings from many randomized control trials conducted in different countries. Currently SFP is possibly the most replicated family skills training program in the world by independent research teams and many local agencies with effectiveness or translational research results that are often larger than the prior federally funded efficacy or randomized control trials (RCTs) [15]. Steps to cultural adaptation of evidence-based family interventions are also reviewed and the research results of SFP cultural adaptations for different ethnic groups in many countries. These results found an average of 40% better recruitment and retention rates for the families in five phase-in studies with major ethnic populations (African, Spanish speaking, American Indian, Asian, and Pacific Islander). The culturally adapted versions were implemented in years three to four and then compared to the generic SFP implemented in years one and two [16, 17]. Following the recommended steps to SFP cultural adaptation, many countries in Europe have had amazingly successful implementation and positive outcomes as detailed in a new European Monitoring Centre for Drugs and Drug Addiction (EMCDDA) publication on EBP alcohol and drug prevention replications in Europe [18] as found on their website, http://www.emcdda.europa.eu/publications/thematic-papers/north-american-drug-prevention-programmes.

The fourth section is dedicated to recommendations for the future to prevention substance abuse and other impulse control disorders including ideas for faster dissemination via computers or mass media such as web-based programs, You Tube, and television.

## 2. Part 1: Substance Abuse Rates Worldwide

Substance abuse has been a concern for many years, but recently there have been alarming rates of increasing adolescent tobacco, alcohol, and drug use worldwide. In many industrialized countries, adolescent legal and illegal substance use has been rising for the past five years [19–21], along with concerns about youth risky consumption patterns in Europe and the USA [22–24]. The biggest recent increase in adolescents' substance use has been in use of party drugs—marijuana, prescription drugs, and binging on alcohol. Not only are adolescents suffering from substance abuse, but also from problems with the law related to underage tobacco and alcohol misuse and illegal drug abuse. The increasing aggressive and defiant behavior, present in young children and teens, demonstrates the need for implementing parenting and family programs to help families raise their children in a more secure and responsible environment [9]. By intervening early in life, these efforts aim at turning developmental pathways away from chronic substance use, delinquency, and health care, thus elevating the financial burden to the public sector caused by children, adolescents, and young adults with substance use disorders [25].

*Consequences of Adolescent Substance Misuse*. Regular illegal substance misuse in adolescents and substance use disorders (SUD) are related to physical and mental health disorders, especially when consumption begins early in adolescence. Youth who begin regular substance use before 15 years of age are at much higher risk of later addictions, and increased risk of diseases of lifestyle (e.g., type 2 diabetes, obesity, liver disorders, HIV/ADS, sexually transmitted diseases, cancer, and heart attacks) and mortality. Chemically altering brain chemistry has been found related to decreased brain functioning (learning, expectation, motivation, and memory) by damaging neurotransmitter balance particularly for dopamine and serotonin. Brain PET scans find that it can take at least two years for the brain functioning to return to normal after using cocaine [26]. Since repeated use of uppers can blunt dopamine receptors, this helps to explain why it is so hard for recovering addicts to find any pleasure in normal activities and the intense craving for the drug to experience pleasure again.

*Economic Costs.* Physical and behavioral health care costs are higher in people with substance use disorders. Health care costs have been estimated to be increased by as much as 50% because of the health consequences of alcohol and drug abuse. Unfortunately, the costs of alcohol and drug abuse are increasing rapidly with increasing use rates in adolescents, particularly very young preteens. In USA, between 1995 and 2000 the estimated economic costs of substance abuse increased from $278 billion to over $366 billion comprised of $181 in illegal drug costs and $185 for alcohol misuse costs. The costs rise to $560 billion if the $193 billion in tobacco use costs are included [27]. However, even with the reduction in adolescent use from about 1998 until 5 years ago, the most recent estimate for illegal drug use alone in 1997 which is the most recent study was 197 billion or an increase of $16 million in economic costs [28]. However, these costs only include illicit drug use and not the higher cost of alcohol abuse. These economic costs include health care and treatment costs as well as lost earnings because of premature death, unemployment, and impaired productivity as well as criminal justice activities such as law enforcement and incarceration costs. In 2013, the US federal budget request alone for substance abuse treatment, prevention, law enforcement, interdiction, and internal costs was over $26.5 billion [29]. This 2013 budget included new federal funds for prevention services for children at risk of child maltreatment because of living with drug abusing parents. Estimates for cost of FASD are hard to find, but Popova and associates [30] estimated that the lifetime economic costs of just one baby born with fetal alcohol syndrome in 2002 was estimated at $2 million adjusted for inflation from the 1980 cost estimate of 588,000. The total economic cost for the U.S. was about $5.4 billion in 2003. Additionally substance abuse by parents is a major factor in child abuse and neglect contributing to the high cost of child protective services and long-term foster care. In a time of economic downturn countries cannot afford this staggering loss, which was almost as much as the annual US budget shortfall of 642 billion in 2013.

### 2.1. Incidence and Prevalence of Substance Abuse

*Substance Use by Countries*. Among 17 nations surveyed by the World Health Organization [31] the United States ranks first in lifetime use of three substances—cocaine, cannabis, and tobacco—and is in sixth place for alcohol use. According to Table 1, sixteen percent of US respondents (ages 15–21 years) said they had ever used cocaine, as compared with about 4 percent of people surveyed in Colombia, Mexico, New Zealand, and Spain. Rates of lifetime cocaine use dipped much lower in the other nations. For cannabis, New Zealand is the only nation to nearly match the US rate of 42.4 percent. Lifetime tobacco use in USA is 73.6 percent, with Lebanon next, at 67.4 percent.

*Gender Differences in Adult Substance Use Rates.* Worldwide, WHO researchers [31] report that men are more likely than women to use cocaine, cannabis, tobacco, and alcohol. The nonmedical use of tranquillizers, sedatives, and prescription drugs is more prevalent among adult females than adult males. In South America and Central America, for example, lifetime prevalence is 6.6% for females and 3.8% for males, while the corresponding prevalence rates in Europe were 13.0% for females and 7.9% for males [32].

*Gender Differences in Adolescent Substance Use Rates.*  The rates of annual illicit drug use for high school seniors, while peaking in 1999 at 42.1%, had decreased by 2007 to 35.9%; however, it has increased in the past four years to 40% [21]. These increases are notable, but marked differences in gender persist. This gender gap of boys using more has disappeared in younger cohorts except for heavy use. In fact, in nearly all drug use categories girls have increased more, decreased less, or maintained essentially the same rates of use for the past two decades. An unexpected phenomenon was that young girls began using illicit drugs more than boys beginning in the early 1990s. By 1995, 8th grade girls exceeded boys in their use of cigarettes, methamphetamines, amphetamines, cocaine, crack, inhalants, and tranquilizers and by 2002 in 30-day alcohol use. By 2005 this same cohort of 10th grade girls exceeded boys in 30-day alcohol use until a slight turndown beginning four years ago.

In general, boys use illicit drugs and alcohol with greater frequency. The annual illicit substance use rates including marijuana for 12th graders show that girls' use was slightly lower than boys' for some time and in 2012 girls reduced their use more than boys by 1.1% to 35.1% compared to 43.5% for boys. The annual illicit drug use rates for 10th and 8th grade girls have always been slightly lower than for boys except in 1992 and 2005. However, in annual illicit drug use excluding marijuana, 8th grade girls have always exceeded boys' use that now stands at 6.0% for girls and 4.8% for boys. By 2006 adolescent girls slightly exceeded boys in substance dependence or abuse (8.1% versus 8.0%) as measured by regular problematic use indicative of needing treatment in the past year. In 2010, the rates were still 7.7% for girls and 6.9% for boys 13 to 17 years old [33]. The use of stimulant drugs or uppers became very popular in young girls to reduce weight and self-medicate depression such that girls have continued their nearly fixed trend of outpacing boys in the use of amphetamines and amphetamine-type substances in all secondary grade levels, with 5.7% versus 3.5% for 8th graders, 8.9% versus 6.7% for 10th graders, and 8.5% versus 7.4% versus for 12th graders for girls versus boys, respectively [21]. Young girls may have unique risk and protective factors related to the breakdown of the family, depression, and increased desire for thinness [34].

NIDA's Dr. Compton suggests that one reason for the higher US lifetime rates might be because drug-use epidemics in USA preceded those of other nations by a decade or more. In the 1970's epidemic, adolescent drug use increased in marijuana, LSD, and heroin mostly in boys. Drug use gradually declined from 1980 to 1992 when drug use rates increased dramatically with a 200% increase in marijuana use and a 500% increase in heroin use between 1992 and 1997. Most of this increase was due to increase in drug use for girls. In other countries, use in adolescent girls has likewise been increasing in the last decade to the point where alcohol or drug use is higher in girls than boys. Tobacco use rates in US youth have been declining since 1991. Likewise, alcohol use rates have been declining in US youth since 1975, however, not so in Europe. For instance, Irish girls have higher regular alcohol use rates than boys. In the UK, binge drinking in girls was a major concern in the mid-2000s. Hence, the need for effective prevention programs and policies for girls became critically important. Both countries have invested heavily in evidence-based school and family-based prevention programs with use rates in girls declining in the past few years.

These reductions in alcohol and drug use in US teens are counterbalanced by increases for four years in use of *any illicit drug* due largely to increased use of marijuana—the most widely used of all the illicit drugs. In 2011, 50% of high school seniors reported having tried an illicit drug at some time; 40% used one or more drugs in the past 12 months, and 25% used one or more drugs in the prior 30 days [20]. The US policy has been to cut prevention funding to increase funding for drug treatment programs, with these dire results.

Speculations concerning reasons for increased use in girls include increased (1) opportunity for girls to use, (2) norms for increased equality in women even in substance use, and (3) the breakdown of the family that appears to impact girls more than boys [34].

### 2.2. Comparisons in Youth Drug Use Trends in Europe and USA (ESPAD and MTF)

In 2011, more than 100,000 students from 36 countries took part in the European School Survey Project on Alcohol and Other Drugs (ESPAD). Since ESPAD is similar to the United States Monitoring the Future Survey (MTF) comparisons for 15 and 16 year olds can be made and include the following.

- Fewer teenagers in the United States smoke and drink compared to their European counterparts, but more use drugs.
- 27% of US adolescents had consumed alcohol in the past month compared to 57% of Europeans with 87% having lifetime use rates. These rates increased significantly in 2011 in European teens. However, young US girls aged 13 to 15 years drink more regularly than do boys, probably because of associating with older boys [34].
- 12% of American teens had smoked tobacco, compared to 20% for the Europeans. However, only 8.6% of boys and 8.1% of girls smoked in last 30 days [35] compared to 28% in Europe [36].
- 18% of American teens had used marijuana or hashish compared to 17% of European teens. Only teens in France (24%) and Monaco (21%) had higher use rates.
- American teens had higher other drug rates, such as LSD, ecstasy, and amphetamines—at 16%, compared to 6% across Europe.

### 2.3. Gender Differences in US and EU Teen Drug Use

*USA.* In 2010, as in prior years, the rate of *current illicit drug use* among US persons aged 12 or older was higher for males (11.2%) than for females (6.8%). Males were more likely than females to be current users of several different illicit drugs, including marijuana (9.1 versus 4.7%), nonmedical use of psychotherapeutic drugs (3.0 versus 2.5%), and current nonmedical users of pain relievers (3.0 versus 2.0%), cocaine (0.8 versus 0.4%), and hallucinogens (0.6 versus 0.3%) [37]. Drugs showing some evidence of declines in adolescent use in 2010 include inhalants, cocaine powder, crack cocaine, the narcotic drug Vicodin, the stimulant drug Adderall, sedatives, tranquilizers, and over-the-counter cough and cold medicines used to get high. The proportion of 12th graders indicating that they have used *any prescription drug outside of medical supervision* in their lifetime, or in the last year, has remained quite stable since 2007.

*Europe. *The average figures for lifetime, past 12-month and past 30-day *alcohol use* prevalence are about the same for boys and girls, but for more frequent drinking within each time frame, boys have higher consumption. Boys in most EU countries drink about one-third more than girls (2011 averages of 5.8 versus 4.3 centiliters of 100% alcohol). However, in a couple of countries (Iceland and Sweden) the average quantities were about the same among girls as among boys. In a large majority of the countries, beer is dominant beverage among boys. Spirits are the most important beverage among girls in just over half of the countries [36].

The *“heavy episodic drinking”* in the past 30 days in girls has increased dramatically increasing from 29% in 1995 to 41% in 2007 and 38% in 2011. In UK club hopping and binge drinking became a national concern when girls were spending most of their money on alcohol. While among boys these alcohol binge drinking rates are slightly higher in 2011 (43%) than in girls, it has remained relatively stable since the 1995 rate of 41% [36].

However, alcohol-related problems are more common among boys in terms of physical fights and trouble with the police. Fight injuries have become so much of a problem in UK pubs that large glass mugs have been banned. The popularity of club binge drinking and club drugs has been spreading to other EU countries where UK teens spend their vacation including Spain, Portugal, and Italy [36].

There were small gender differences in 30-day* cigarettes use* in 2011. At the aggregate country level, the sex differences in 2011 were negligible for smoking in the past 30 days. So girls are smoking more since in 1995 and 1999 when slightly more boys were smokers. However, in 2011 some individual countries had large sex differences with higher figures for girls in Bulgaria, Monaco, France, Slovenia, Faroe Islands, and Ireland and higher figures for boys in Albania, Cyprus, and Moldova, Ukraine, and Montenegro.

*Illicit Drugs.* In European teens, [36] ESPAD reported use of illicit drugs varies considerably across the countries with higher lifetime experience reported by boys than girls (19% versus 14%). Illicit drug use was significantly higher for boys in 27 countries, with a large gap between the top countries prevalence in Czech Republic (43%), France, and Monaco (about 39%) and lower prevalence rates often found in south-eastern Europe and among Nordic countries. Boys report higher figures in more than two-thirds of the ESPAD countries in 2011.

*Cannabis.* In general, marijuana use has been increasing in adolescents in Europe since 1995 except for a slight downturn since 2007 in some countries. Regular 30-day marijuana use is higher in boys than girls, but it varies considerably by country with highest rates of use in Spain, France, Italy, and UK [19]. Lifetime use of cannabis was reported by more boys (19%) than girls (14%), and the figures were significantly higher for boys in 27 countries. Only a few countries in Europe such as France and the Czech Republic reported higher lifetime use rates than US (35%). Of interest is the fact that the equal rates by girls probably contribute to the high overall teen use rat in France. Past 12 months use was reported by 15% among boys and 11% among girls, while 30-day use was reported by 8% of the boys and 5% of the girls [36] ESPAD.

*Other Illicit Drugs*. In Europe as in USA there has been an increase in adolescent use of designer drugs because of increased production in China, India, Mexico, and internally in-country labs. Online shops have increased in Europe from 170 in 2010 to 693 in 2012 [19] increasing availability for teens. More boys than girls have tried illicit drugs other than cannabis, 7% versus 5% in 2011 [36]. However, more girls (8%) than boys (5%) report nonprescription use of medical drugs. Lifetime use of tranquillizers or sedatives without a doctor's prescription, together with mixing alcohol and pills, is the only substance-use behaviors that have been more common among girls than boys [36]. The use of inhalants increased in both sexes to 10% in 2011 for the first time.

## 3. Part II: Etiology of Adolescent Impulse Control Disorders and Drug Use including Risk and Protective Factors

*It's a Family Disease. *This section of the paper will review the etiology or causes of impulse control disorders with an emphasis on substance abuse. The major conclusion is that the most salient causal factors or mediators are family genetic and environmental influences. It has long been recognized that substance abuse addictions run in families—a lesson that the obesity prevention researchers need to pay attention to. The early longitudinal studies like the Harvard Study [38] and twin and adoption studies [39] suggest that there is a large genetic contribution to the addictions and alcohol abuse.

Children of substance abusers that have highest risk for later addiction are those who are family-history positive (FH+) for Type II alcoholism, characterized by early onset alcohol use. Schuckit's [40] research found that they manifest the phenotype of the “Over-stressed Youth Syndrome” (as labeled by Kumpfer in her 1987 review of risk factors for the National Institute on Drug Abuse (NIDA)). Schuckit conducted research with college students with a large number of male relatives with early onset alcoholism. He found they have rapid brainwaves, increased emotional labiality and ANS hyper reactivity. Consuming alcohol smoothed out their brainwaves and emotional reaction. In Type 2 alcoholic families, characterized by early onset of addictions particularly in males, the increased risk is 18-fold.

In a 60-year longitudinal study of a sample of Harvard University graduates including President Kennedy and a sample of inner city Boston men, psychiatrist George Valliant [38] found that the higher prevalence of alcohol abuse was primarily found in those of Northern European ancestry as compared to those of Southern European ancestry. Later studies of identical and fraternal twins, who were adopted to different families in Sweden, Denmark, and USA, found that if one identical twin developed alcoholism, there was an 80% chance that the other twin living in a different family environment would also become an alcoholic. However, this genetic risk was half as great in women at only 40%. This could suggest greater environmental protective factors for women or girls or that some of the genes related to addictions and ICDs are on the sex-linked chromosome.

These genetic risks help to explain why addiction appears to be a “family disease” with children of parents with substance use disorders at much higher genetic risk (for a literature review of children of substance abusers risk and protective factors see [41]).

*Epigenetics: The Gene x Environment Interaction.* For some time it has been clear that there is a gene by environment interaction as found in longitudinal large population studies like the Dunedin Study in New Zealand [42]. Identical twins can develop differently and even have different hair and eye color despite having identical genetic codes. We all know we have a genome, but recently discovered is the fact that we all have an epigenome. The epigenome is the way that the strands of DNA are wrapped around protein bodies. The more tightly wound because of stress, the more likely a person is to express genetically inherited diseases.

Epigenetic research studies the influence of the environment on the epigenome and genetic expression. Recent studies mostly with mice suggest that nurturing parenting could be one of the most critical variables in protecting people from manifesting unhealthy genetic diseases. The direct mechanism is reducing stress and cortisol that can turn on inherited genes. Hence, another reason to improve parenting skills is to reduce family risk factors that contribute to the high rates of substance abuse in youth today.

This epigenetic research suggests that positive parenting and family functioning that reduces stress can reduce the manifestation of genetic predisposition. Researchers at McGill University [43] suggested that maternal stress and fetal undernutrition *in utero* leading to low birth weight can result in poor birth outcomes that predict poorer health over the lifespan. Lack of a nurturing parent can program increased stress reactions in children resulting in reduced exploratory behaviors, cognitive development, and oxytocin binding even in later generations [44, 45]. This highly genetic type of substance abuse was also called Male-limited Alcoholism since it primarily impacted males. Kumpfer [46] has hypothesized that many of the genes that promote alcohol and drug use are on the sex-linked chromosome. Hence, females have to inherit risky genes related to substance use from both sides of the family to manifest the disease, but male from only one side. Hence, it would make logic sense that females should have only half the prevalence of substance abuse. However, now with epigenetic research and the epigenome, we know that there are many genes by environment interactions that can actually modify gene expression and also gene transmission to the next generation. Nurturing parenting and family support that reduces cortisol levels appears to be a major protective mechanism in the phenotypic expression of genetic family history risks for substance abuse and other costly health conditions.

*Short Alleles of the Serotonin Transporter Gene Increases Genetic Risk for ICDs.* While there are likely many genes involved, researchers have found that genetically at-risk adolescents are those with only one or two short alleles of the 5-HTTLPR serotonin transporter gene. They are more likely to become substance abusers [47, 48], depressed [42], or delinquent with lower behavioral and emotional control [49, 50]. However, Brody and associates [51–54] have found that if their family attended an African American adaptations of the *Strengthening Families Program* (SFP), called *Strong African American Families*, they had reduced risk. They used saliva samples from the students in the study five years after SFP involvement to test for this risky gene variant. In this 5-year follow-up study at age 17 of years of a school-based RCT found 50% less 30-day alcohol use, depression/anxiety, delinquency, and sexual intercourse rates. Of interest is the fact that youth (about 40% of the European but 60% of Asian population) with this short serotonin transporter gene are also more sensitive to stressors that could lead them to self-medicate with alcohol and drugs. Also, alcohol increased serotonin transport in the neuronal synapse.

*Prevalence of Children of Substance Abusers.* About 10.5% of US children currently live with a parent who had a diagnosed alcohol use disorder, but about 25% of US children (19 million) have been exposed to parental alcoholism at some point while growing up and about 12.7%, or 9.2 million have been exposed to parental drug abuse [37]. These children might have been damaged by alcohol or drug exposure *in utero* or impacted by a chaotic and nonsupportive family environment [55]. Studies of the child's perspective toward their alcohol or drug abusing parents found three common themes: family role reversal, keeping the family secret, and coping strategies [56].

*Children of Parents with Substance Use Disorders Are at Higher Risk for Addictions.* Research suggests that children of addicted parents experience two to nine times greater risk of becoming substance abusers as adolescents or adults [57] despite positive and adaptive behavioral outcomes of many of these children. They are also more likely to initiate drinking at an earlier age and escalate more quickly to SUDs. The risk for later SUDs depends on the degree of risk factors compared to protective factors including the extent of their family history of alcoholism (FH+). Factor to consider are whether one or both parents are abusers, the addiction severity and duration, and the type of alcoholism that runs in the family, such as the highly genetic Type II alcoholism or lower risk Type I environmentally caused alcoholism as well as the extent of parents' antisocial behavior, and health and mental health problems [40]. Because of their higher risk, children of substance abusers need to have family-based prevention services provided to their families and also extra community support services [41]. They are at higher risk also for child maltreatment. Provision of family-based prevention services that are particularly designed for children of drug abusers, such as the author's *Strengthening Families Program,* has been found to reduce days in foster care in half in a five-year multisite federally funded study in Kansas [58].

*Family Environmental Impacts: Global Negative Impact of Adverse Childhood Experiences (ACEs)*. A decade long study by the Centers for Disease Control and Prevention (CDC) of members of a health management organization (HMO) found that higher health costs and health problems were associated with multiple adverse early childhood circumstances [59] and parental substance abuse. If both parents had SUDs, the risk for later adult alcoholism also increased dramatically. The mean number of ACEs for persons with no parental alcohol abuse was only 1.4 compared to a mean of 3.8 ACEs in children with both parents having SUDS.

*The Gender Gap and Recent Increases in Girls Use of Drugs*. Since our culture puts different expectations on girls than boys, gender would logically play a part in differential risk and protective factors for smoking and other substance abuse. The historic pattern of inattention to substance use and misuse, delinquency, and mental health problems in girls and women has recently been changing. Girls are attracting attention as more enter drug treatment and are mothers. They become addicted more quickly and for different reasons. They appear to be influenced more by pressures to use or by observing the use of substances by friends, peers, and family members. But currently, the rise and the reasons in drug misuse in girls remain unclear.

Since there is no single factor that puts girls “at risk” for problem behaviours such as substance use and delinquency, there are many possible causal mechanisms that have been hypothesized [60, 61] related to differences in genetic and environmental risk. Some researchers are beginning to search for distinctly female etiological patterns of substance use and misuse and delinquency.

Several explanations for gender gap in substance use have been advanced including the increased genetic risk in males and recent environmental risk in girls with rapid changes in social roles. Biological and socially constructed gender differences produce unique development trajectories for males and females, with concomitant risk, resiliency, and protective factors that lead to different substance use behaviours and different motivations for using substance [62, 63]. The major mediating factors appears to vary mostly in the strength of their impact on girls as compared to boys.

### 3.1. Tested Causal Models (Social Ecology Model)

Because there are so many risk and protective factors for drug use found in individual studies, an overall tested model ordering these mediators and moderators was needed. Luckily, Swedish researcher developed advances on path models called Structural Equation Models (SEM) that allowed researchers to test the strength and ordering of many different causal factors if they have a large dataset including most of these factors.

These tested causal models suggest that the major causal factors are peer values and norms as the final pathway to drug use, but also that youth are less likely to associate with drug using friends if they are close to prosocial, nurturing, and nondrug using parents. These models discovered three family protective factors, namely, positive parent/child relationship, parental supervision/monitoring, and consistent discipline, and parental communications of nondrug use expectations and family values are the major reasons youth do not use drugs or engage in other adolescent behavioral problems [5, 64, 65].

*The Social Ecology Model of Adolescent Vulnerability of Substance Abuse.* The first SEM test of this model [66] did found no major differences in the causal pathways to drug use or delinquency in girls compared to boys, but because the sample size was only 800 youth, the single family factor was just called “family environment.” When Dr. Kumpfer became the Director of the SAMHSA Center for Substance Abuse Prevention (CSAP), she found a database of 10,000 youth from all over the country with multiple ethnicities. When this new model was tested [6, 7] the final pathway to drug use collapsed into a combined parental norms and peer norms factor because of the close association.

**Figure 1:**
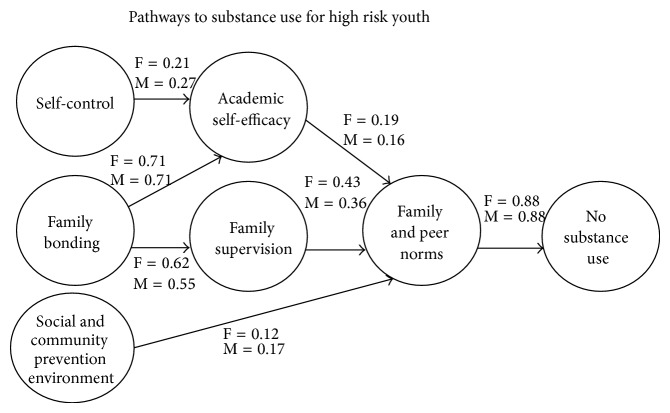
Social Ecology Model of Substance Abuse by Gender (males/females).

*Gender Differences in Precursors.* As shown in Figure 1, the final pathway to drug use of parent/peer influence was equally powerful for girls and boys. However, family bonding and parental supervision have a slightly greater impact on girl's choice of substance-using or non-using friends. Behavioral and emotional self-control had a slightly larger role in later drug use in boys, possibly because boys seem more prone to difficulties in this area. Girls were more influenced by their academic performance and self-efficacy than boys. The community and neighbourhood environment has a greater influence on boys than girls. Never predicted was the strong pathway between family bonding and academic performance that seems logical.

A similar SEM model was tested for school failure, delinquency, and teen pregnancy as well as alcohol and drug use with similar results [5, 67] finding that low parental supervision had a greater influence on adolescent girls' alcohol and drug use than on boys. Also in this sample of African-American youth, school bonding has less of a protective factor in girls than for boys.

In conclusion, these tested theoretical models provide suggestive evidence that girls are more influenced by family protective factors than are boys. Girls are more adjusted and dependent on a positive relationship with their parents and friends to define their self-worth. On the contrary, girls appear to be slightly less influenced by their community environment than are boys.

### 3.2. Risk and Protective Factors in Girls

The rest of this section of the guidelines on causal factors will address risk and protective factor research organized by the Social Ecology Model Framework. It will start with the most influential factors, peer and family influence, behavioural control, school performance, and then community norms and values.

*Gender Sensitive Risk Factors.* In recent presentation in June 2012 at the NIDA Women's conference, Dr. Cora Lee Wetherington summarized a more detailed list of risk factors for girls and boys. As can be seen in Table 2, girls were cited as having greater sensitivity to family problems.

The higher predictive power for boys than for girls of depression, peer difficulties, and aggressiveness in the first grade could be related to the greater influence of behavioural self-control problems in boys substance use. However, understanding why conduct disorders are more predictive of drug use in girls is harder to explain except by genetic risks.

*(1) Genetic Risks.* Genetic risk is pretty constant although there is recent epigenetic research suggesting gene/environment interactions due to maternal stress that can cause changes in genes *in utero* that maybe passed into the next generation [45]. Hence, these short-term prevalence increases in girls use are likely due more to the significant changes in social environments and social roles for women. With increased access to jobs, affluence, and social freedoms, young girls have begun to use more. Kumpfer and associates [34] called this the “Virginia Slims Effect” related to sexual and social role liberation. In developing countries, one major cause for increased drug use in girls relates to “differential generational acculturation.” This phenomenon is the difference in cultural values, lifestyle, clothing, language, and traditions between teens, their parents, and grandparents. In a tested SEM theory in Thailand, Rodnium [11] found that differential generational acculturation led to increased family conflict with teens rejecting traditional family values, breaking away from the family influence, sometimes leaving home for cities and increased freedom to use alcohol and drugs.

One major reason for increased heroin and other drug use in girls is sex-trafficking that is prominent in Southeast Asia and Eastern European countries. Girls in some cases have to leave home for economic reasons, being told they will be maids or housekeepers in city hotels, only to find they were sold into prostitution. Some girls like this lifestyle because they can earn large sums of money for clothes and entertainment, plus to be free of parental influence. In Russia, one of the most desired occupation by girls is to become a prostitute. Unfortunately, this lifestyle is traditionally associated with heavy drug use like heroin even in USA and Europe.

*(2) Family Risks and Protective Factors.* Without extended family protection and family or agency support, many children of substance misusers live in disruptive family environments. These environments are frequently characterized by family conflict, disorganization, or disrupted family rituals (i.e., inconsistent meals together, bed time rituals, holidays, etc.). The environment contributes to an already elevated sense of anxiety and stress in these children.

As suggested by the Social Ecology Model discussed earlier, girls are more impacted than boys by the quality of the family environment. Parent/child attachment or bond impacts parental monitoring and supervision of youth's activities and amount of time spent teaching positive values and expectations for behaviors. Hence, it is hypothesized that the breakdown of the family in recent years has contributed substantially to the large increase in adolescent girls rates of alcohol use, illegal drug use, and delinquency.

Because of worldwide economic strains, parents are working more and spending less time parenting and being involved with their children. The Annenberg Center [68] estimates that in USA the amount of time parents spend with their children decreased from 6.2 hours to 4.5 hours between 2005 and 2008. Few parents in USA still have a family meal, each day, with all of their children; although two-thirds of children in other countries still have the main meal with their parents [69]. However, fewer children talk with their parents on a regular basis. Living with drug addicted caretakers who spend an average about half as much time with their children only increases children's stress levels and feeling of lack of protection. Research has shown that families that maintain certain “rituals,” can help mediate the stress and chaos of addiction. Sober parents who are able to provide stability, support, and nurturing also help minimize confusion and strengthen children. Sometimes family life is less damaging because children rely on “adaptive distancing,” a technique in which the child separates from the “centrifugal pull” of family problems in order to maintain meaningful pursuits and seek fulfillment in life, school, and friendships [70].

This lack of parental attention seems to be more detrimental to girls than boys; hence, the need for family strengthening interventions that encourage parents to be more involved with their children. Also demographic and social changes in the last three decades have resulted in families where children have a higher probability of having a single parent, being part of a stepfamily or foster family, and experiencing parental separation due marital discord, military services, jail time, or parents working in another location. The impact this experience has on children is a key issue for policymakers since although the government wants to support stable relationships between parents, where they break down there is a responsibility to provide support to optimize positive outcomes for children.

It has been also hypothesized that family breakdown has a greater influence on women's expectations of relationships in adulthood [71]. Cohort studies in the UK also indicate that young women are at greater risk of educational underachievement, leaving school early, lower occupational status, and of early family formation and dissolution [72].

Some longitudinal studies [73], including the National Longitudinal Adolescent Health Survey [74] that accompanied preteens during periods normally associated with drug use, found that despite the factors associated with peers becoming more important among the preadolescence and adolescence, parental factors were still the most influential. These findings are replicated in many other US youth surveys, including Monitoring the Future school survey [75], SAMHSA, the Parent's Resource Institute for Drug Education (PRIDE) survey that concluded that most youth who use alcohol or drugs do it in homes, which can be prevented by family monitoring, supervision, and attention given the availability of alcohol and drugs at home.

Despite these data, most prevention programs continue to neglect the family as the primary target for enhancing protective parenting and relaying messages to parents about what they can do to better protect their children from substance abuse and other impulse control disorders.

## 4. Part III: Strengthening Families Interventions to Prevent Substance Abuse and Other Impulse Control Disorders

### 4.1. Theoretical Justification for Family Interventions

This first section will provide a review of Bowen's Family Systems Theory and the notion of changing the full family system that provided the major reason that family interventions work well. Bronfenbrenner' Social Ecology Model Theory, and Bowlby Attachment Theory also provide a justification for working with the family. These theories are discussed below as well as some family therapy interventions that were the early prototypes based on these family theories.

#### 4.1.1. Bowen's Family Systems Theory

Probably the major impetus for a focus on improvements in the whole family system as compared to individual therapeutic approaches was wielded by psychiatrist and family therapist Murray Bowen. Bowen was largely responsible for formulating the backbone of family systems theory and family therapy, mostly by encouraging therapists to include a broader natural scientific perspective in treating psychopathology. Bowen strongly believed that the family should be discussed as a functional system, rather than a collection of individuals. In other words, the unit of treatment should be the family rather than single identified members. He postulated that a family member's behavior can only be understood in relation to other family members and the various social contexts they create [76]. Psychopathology and addiction reflected an “imbalance in functioning in the total family system” [77, page 97]. Corrective actions require modification in patterns of functioning and this notion that the entire family needs to be involved in a therapeutic healing would pave the way for later family-based prevention programs to incorporate intervention strategies that involve both the parent and child.

The prior neglect of treating the full family system owed much to the dominance of Freudian thinking with its emphasis on individual psychopathology, the ontogenesis of personality, and the curative power of individual psychotherapy for treating mental health problems. In trying to get scholars and clinicians to recognize the importance of family systems in mental health, Bowen invoked the analogy of a jigsaw puzzle, suggesting that the puzzle is not complete until we can see the whole picture. His clinical work with schizophrenic patients revealed the closely intertwined nature of family emotional ties and psychological boundaries that existed between primary caregivers and the patients he was treating.

Salvador Minuchin developed *Structural Family Therapy* based on Bowen's family systems theory based after his disappointing clinical experiences in treating adolescents individually in his inpatient clinic. He would see improvements in symptoms, but the adolescents regressed and symptoms returned upon their return to their home life with their families [78]. Similar to Bowen's family systems theory, problems in the adolescent's functioning was hypothesized to be rooted within the family system and not any one particular member of the family [78]. Minuchin further hypothesized that chronic relationship issues within and between the family members and inappropriate relationship boundaries within the family and not just problems resting with the adolescent were the major contributors to the teen's maladaptive behavioral patterns [79]. His new therapy techniques, instilled in structural family therapy, were designed to collaborate with the family to create a more functional and satisfying family system. There need to be appropriate boundaries between parents and children, so parents can exert an appropriate amount of power and control within the household. The parent/child boundary can be corrupted if children become parentified and are expected to perform parent-like responsibilities within the household. This problem occurs frequently in single-parent households, or when parents are alcoholics, drug abusers, or mentally ill and depressed and where there are many children, some of whom are much older than others in the household. Role reversal can also happen in immigrant and refugee families where older child learn the language much faster and take on the parent role.

#### 4.1.2. Bowen, Brofrenbrenner, and Bowlby

Bowen's theorizing about the primacy of the family on children was also supported by other etiological or causal theories such as Bronfenbrenner's social ecology model [80] that placed the family as the most important influence on the child followed by more distal influences such as friends, school, community, and media. In addition, Bowlby's [81] conceptualization of attachment highlighted the primordial bond between caregiver and infant. A lack of early parent/child attachment was used to explain later vulnerability and psychopathology. Indeed later research supported the association between *reactive attachment disorder *(RAD) and poor social adjustment, lack of trust, and emotional feelings for others that can lead to violent crimes. In developing his model of attachment, Bowlby relied on a class of social behavior designed around connections to and separation from mother to distinguish normal from traumatic development. According to Bowlby, attachment schemas or “internal working models” lay the foundation for emotional development, providing outlets for our feelings of anxiety, happiness, sadness, or confusion.

Bowlby's attachment theory [82] has tremendous parallels with Bowen's concept of family systems theory although Bowlby placed increased emphasis on the critical importance of secure attachments. However, Bowen also stressed the fact that a primary goal of childhood is differentiation of the self (child) from parent that happens through secure attachment. Clinicians have found that children of substance abusing parents are more likely to suffer from insecure attachment because of neglect or abuse. Some have used the Ainsley Stranger Situation Test to determine if an infant or toddler is insecurely attached to a caregiver. One of the most effective therapeutic treatments to improve parent-child bonding and treat RAD according to research by Egeland and Erikson [83] is “special play” developed by Kate Kogen.

Of interest for this chapter is the fact that special play, also called “Child's Game” in several of the most effective family-based prevention programs, is a foundational basis of most behavioral skills training prevention and therapy programs (e.g., *FAST Track, Strengthening Families Program, Families and Schools Together, Parent Child Interactive Therapy,* and *Incredible Years* to name a few). This technique teaches a parent to allow a child or teen to select an activity for them to do together to increase attachment. Parents are coached to use a nonjudgmental running dialogue (like a sportscaster) of what the child is doing to show positive attention. They are not to take control by asking questions, teaching, criticizing, or suggesting new activities. This special play is different than play therapy where the therapist takes control by asking the child why they are doing certain types of play activities. This nondirective play is critical to developing bonding.

#### 4.1.3. The Development of Family-Centered Interventions

Researchers that began to construct family-based interventions took into consideration the growing sentiment by Bowlby, Bowen, Minuchin, and others that children's problems are rooted in the way parents deal with or treat their children. However, they needed effective intervention methods to improve family bonding, communication, organization, and reduce conflict. The inspiration for these effective techniques came from B.F.Skinner's operant conditioning techniques that were misunderstood and much maligned in the 1970s as inhuman but were redeemed by Bandura at Stanford University with his social learning theory or cognitive behavioral theories and self-efficacy theories [84]. Teaching parents to use positive reinforcement (attention, praise) for wanted behaviors and ignoring unwanted behaviors was developed into highly effective clinical methods by Gerald Patterson at the University of Oregon. His cognitive behavioral change theories or skills training [85], developed to reduce psychopathology in children and families, became the basis of most of the effective parenting and family skills training programs listed as evidence-based family prevention and treatment programs including the ones listed in this chapter. His family techniques were designed originally for individual families in clinics, but later, along with Marian Forgatch, he developed a group based version [86].

These family-focused interventions proved to be particularly effective in reducing drug use and intermediate risk factors, such as conduct disorders, aggression, and family conflict, as well as improving protective factors such as social competencies, peer resistance skills, family and school bonding, school performance, and family organization and cohesion [6].

*Types of Family Interventions and Effectiveness. *The first review supported by the US government to determine EBP approaches or types of family interventions was cochaired by the main author and Dr. Jose Szapocznik. It was determined that four family-based approaches demonstrated the highest level of evidence of effectiveness in reducing behavioral and emotional problems in children five years old and up. These evidence-based family intervention approaches, described in more detail by Kumpfer and Alvarado [4], include (1) behavioral parent training (primarily cognitive/behavioral parent training); (2) family skills training (including parent training, children's skills training, and family practice time together); (3) family therapy (structural, functional, or behavioral family therapy); and (4) in-home family support.

*Searches for Evidence-Based Family Interventions.* For the past 20 years, the author and her colleagues have conducted periodic expert reviews to identify individual evidence-based family interventions. The first review conducted for the federal government (OJJDP/CSAP) of only US programs revealed 35 parenting and family strengthening programs with some level of evidence of effectiveness. Only seven family interventions of these 35 programs met the highest level of evidence of effectiveness, or Exemplary I, which required a minimum of two randomized control trials with positive results implemented by at least two independent research teams with different populations. These Exemplary I family programs include *Helping the Noncompliant Child (the basis of FAST Track project),* The *Incredible Years, the Strengthening Families Program, Functional Family Therapy, Multisystemic Family Therapy, Preparing for the Drug Free Years (now called Guiding Good Choices), and Treatment Foster Care.* Seven programs were classified into the Exemplary II level because they had at least one randomized control trial with positive prevention results. The other programs were classified primarily into the Model Level, because they had only quasi-experimental research results. Some Promising Level programs were added to the list because they were programs that were based on existing proven programs but did not yet have outcome results.

A worldwide review conducted for the United Nations Office of Drugs and Crime (UNODC) in Vienna by the author identified 185 family programs with 60 being promising family interventions, but only 25 finally family EBPs selected for their website and published in the Compendium [87]. An expert review group also contributed to a publication on core contents and steps to cultural adaptation of evidence-based prevention programs [2]. Information on these specific family interventions including program descriptions, web sites, and contact information can be found at http://www.strengtheningfamilies.org and the UNODC web site for the Compendium [87].

*Core Essential Components of Family Interventions.* Miller and Hendrie [14] found in their cost/benefit analysis that particularly strong programs for teen drug prevention are those that are designed to strengthen bonds to family, school, and community and facilitate participant development of skills, rather than just educating participants on the dangers of substance abuse. In fact the most effective drug prevention programs include limited discussions of drug effects or consequences. Research suggests that family skills training, including interactive training such as role playing, group discussion, and homework assignments, is more effective than reading and lecturing [4]. The importance of family practice time was also confirmed by US Centers for Disease Control and Prevention (CDC) meta-analyses, which found that prevention programs including this component were more successful because the skills practice was more natural and generalizable [88]. The following four content components were found to be significant predictors of larger effect size: Emotional Communication, Practicing with Own Child with Family Coach, Positive Interactions with Child, and Consistent Responding. They point out that two of these skills are beneficial because they improve the parent-child relationship, which subsequently improves behavior.

As for the youth and children components, social skills and emotion regulation skills have been found to be the most important skills to target in order to prevent delinquency [6]. These skills create self-reinforcing prosocial behaviors that allow the child/adolescent to bond with positive adults, authority figures, and peers, and through these positive relationships they can avoid delinquency and have more positive life outcomes.

Other factors that increase program success are having a strengths and resilience-based focus, involving fathers, adapting the program to target the needs and cultural sensitivities of the families, having the appropriate intervention dose, and providing incentives and transportation in order to improve retention [6].

*Evidence-Based Family Interventions Criteria. *The prevention programs included in this review were chosen because they are evidence-based and have passed muster at the highest levels of scientific scrutiny in comparative effectiveness reviews, such as the Cochrane Collaborations in Medicine and Public Health [12]; National Institute of Justice (NIJ) Office of Juvenile Justice and Delinquency Prevention's (OJJDP) Strengthening America's Families review, United Nations Office of Drugs and Crime (UNODC) Compendium of Evidence-based Family Skills Training Programs [87], NIDA Redbook, and SAMHSA National Registry of Evidence-Based Programs and Policies [89]. A recent Cochrane Review of drug prevention programs in nonschool settings [13, 90] concluded that only a few family-based interventions (ISFP, PFDY and Focus on Families) plus motivational interviewing have sufficient evidence of effectiveness to recommend dissemination. Multicomponent community interventions did not have any strong effects on drug use.

*Evidence-Based Family Therapy Interventions*. These evidence-based programs (EBPs) included family interventions targeting indicated prevention or treatment with diagnosed youth. Examples of programs that cover this area include all of the more costly family therapy interventions serving individual dysfunctional families by highly skilled and trained professional family therapists, such as *Brief Strategic and Structural Family Therapy* [91] and *Multidimensional Family Therapy* [92] for treating drug abusing youth, and likewise *Multisystemic Therapy* [93] and *Functional Family Therapy* [94] for treating acting out, conduct disordered, or highly delinquent youth. Comprehensive reviews of evidence-based family therapy programs are available elsewhere [95, 96]. Some of these treatments require case management and in-home visits to referred or mandated in-crisis families because of diagnosed youth behavioral health or delinquency charges or severe parental dysfunction, substance use disorders, or child maltreatment.

*Effectiveness of Family Evidence-Based Programs. *Tobler and Kumpfer's [64] meta-analysis results suggested that family programs are nine times as effective in reducing conduct disorders and child abuse as child-only focused programs [97]. Another particular benefit of family skills training programs is their cost effectiveness [10].

Early elementary school parent training or family skills training programs have been found to be very effective in reducing aggression, conduct disorders, attention deficit/hyperactivity, and oppositional defiant disorders, and preventing child abuse, drug abuse, and delinquency [98]. Family skills training programs (e.g., SFP, ISFP, PFDY) appear to have particular promise. This category of program includes parent training, children's skills training, and a family practice session and is generally implemented with groups of families. The magic in these family skills training programs appears to be having the parents and children directly practice the new skills and have homework assignments to bring their new modes of interacting into their home. The next section will discuss the author's own family skills training program that has been found to be the most effective for preventing impulse control disorders.

### 4.2. Principles of Evidence-Based Family Interventions

There are certain characteristics of effective prevention programs, called principles of prevention, which can be used to judge the potential effectiveness of different prevention programs. Both the National Institute on Drug Abuse (NIDA) and the White House Office of National Drug Control Policy (ONDCP) have published lists of principles for substance abuse prevention programs. A broader “review of reviews” approach [99] was used to extract effectiveness principles from research articles on prevention programs in four content areas (e.g., substance abuse, risky sexual behavior, school failure, and juvenile delinquency and violence). Nine program characteristics were consistently associated with effective prevention programs: comprehensive, theory-driven, appropriately-timed, socioculturally relevant, sufficient dosage, varied teaching methods, positive relationships, well-trained staff, and outcome evaluation.

The principles of effective family-based prevention or treatment interventions are similar to those described by Nation and associates [99] in the *American Psychologist *and included in other reviews of family programs [100], namely the following.

*Family Principle No. 1: There Is No One Best Family-Focused Program*. The best family intervention to select for a particular population must consider the characteristics of the families to be served and the unique design differences in family interventions. Factors to consider include that the program must be as follows.

*(1) Culturally and Gender Competent.* In addition, programs should be “socioculturally relevant,” appropriate and if possible culturally adapted EBPs as specified by Kumpfer and associates [16, 17] because they increase recruitment and retention.

*(2) Sufficient Dosage.* Also the family intervention should match the needs or risk level of the families with “sufficient dosage.” For universal prevention for general low risk populations the program can be shorter of about 5 to 10 sessions. However, for selective prevention for high-risk groups there are more needs or risk and protective factors to be covered. Hence, these programs are generally about 11 to 18 sessions. Indicated prevention for youth already manifesting or diagnosed with risk mediators of the problem to be prevented is generally longer than 16 sessions to be maximally effective.

*(3) Match Specific Needs of Families.* Additionally, there are now family prevention programs designed to match specific family needs or for specific populations at risk, such as children of substance abusers, children of divorced parents, children with autism spectrum disorders, children of depressed mothers or parents with posttraumatic stress disorders (PTSDs), children living in foster families [101], youth involved with juvenile courts, or mental health systems, to name some examples.

*(4) Age and Developmentally Appropriateness.* Hence, when selecting a family program consider the developmental appropriateness of the program for the ages of children involved. As pointed out by Sussman [102], prevention programs should be designed to consider the developmental milestones for the child at different ages, cognitive development, and language levels. Below are evidence-based family approaches and interventions that seem to work best for different ages of children.

*(a) Birth to Three: In-Home Family Support Programs.* The evidence-based family and parenting programs most appropriate for early childhood (birth to 3 Years) tend to focus on providing in-home family support services for very high risk parents such as parents already reported for child maltreatment or neglect, very low income and education levels. The reason higher risk families are generally the only families provided this model of family programming is because these are very expensive programs to administer. There are many factors which make this approach unique; families can stay at their own homes without arranging for transportation, child care, or time off from work to attend family groups. Family in-home support programming is also an effective way to implement an intervention program because it is focused on educating the parents at an early stage of the child's development to have the longest positive impact and give the child a good start in life. According to Russel et al. [103], parents are the most consistent caregivers for their children and can respond positively and effectively to their children when given the knowledge, skills, and support necessary. Many in-home family support programs also deal with all family stressors by providing referrals for child respite care, housing, legal, employment, and behavioral health services. By educating parents with valuable information, the parents are able to provide protective factors to their children.

Overall, in-home family support approaches are an effective approach to help parents learn early how to raise their children effectively according to several meta-analyses [64, 104]. The average effect size for the 14 evidence-based In-home Family Support Programs in the Tobler and Kumpfer [64] meta-analysis was a very large Cohen's d effect size of 1.64, which was larger than the other family approaches.

Examples of evidence-based in-home family support programs include: *The Home Instruction Program for Preschool Youngsters program (HIPPY)* a 30-week program to help parents to improve the cognitive level of the child, *HOMEBUILDERS Program* [105, 106] an intensive in-home crisis intervention for families with children who are in danger of being placed in state-funded care with therapists initially on call 24 hours a day, 7 days a week, and the *Nurse-Family Partnership* developed by David Olds for at risk first time mothers where a registered nurse frequently visits in the first trimester but thins visits as needed until the child's second birthday. The results of a 12-year follow-up by Kitzman and associates [107] reported significant reductions in child substance abuse, improved academic tests scores, grades in reading and math, higher GPA, and reduced internalizing disorders. Reduced child maltreatment reports were also found that involved the mother or the child [108]. Olds and associates [109] also reported on the economic savings for the program from their 12-year follow-up of their Memphis sample. Despite costing about $4,500 per family per year, the “government spent less per year on food stamps, Medicaid, and Aid to Families Dependent Children and Temporary Assistance for Needy Families for nurse-visited than control families.” Cost-benefit analyses found a $5.70 cost benefit ratio [110].

*(b) Childhood (3–12 Years: Parent Skills and Family Skills Training Approaches).* ******The intervention approaches that have the most evidence-based programs for this age group are behavioral parent training, family skills training, and behavioral family therapy. Many of these EBPs are spin-offs of Gerald Patterson's clinical work at the University of Oregon with families of children with conduct disorders. Graduate students or professionals studying with Dr. Patterson designed most of the evidence-based programs for this age of children, namely, *Helping the Noncompliant Child (the basis of FAST Track project),* The *Incredible Years, the Preparing for the Drug Free Years (now called Guiding Good Choices), and Treatment Foster Care. *Other family skills training EBPs were heavily influenced by Patterson's intervention ideas such as that of Kumpfer's *Strengthening Families Program, Sander's Triple-P, and Alexander's Functional Family Therapy.*

Most of these interventions involve the whole family or at least one adult in the family and the identified child working individual with the therapist or in multifamily groups for about 7 to 15 weekly two to three hour sessions. Generally family skills training programs are interactive, multisession programs that allow parents to not only learn positive parenting skills, but also practice them while creating a stronger bond between family members. They can be applied to universal, selective, or indicated prevention with the dosage being increased for the higher risk youth and families. Generally family skills training programs encourage any adult who cares for the child to attend the program. This could include grandparents, aunts, uncles, older siblings, foster parents, and hired caregivers [2]. They all used cognitive behavioral change techniques that include instruction, role modeling, direct parent/child practice with therapist feedback, and home practice assignments to enhance generalization of the new parenting practices to the home. Also praise and rewards or incentives for positive behavior change are core elements of these programs.

While all of these programs are rooted in similar theology, they are not all created equally. Looking more closely at *Triple P* will provide greater insight into how family skills training can be an adaptable, effective option for strengthening the protective factors inherent in strong families. Triple P stands for “*Positive Parenting Program,*” and was developed by Matthew Sanders and fellow colleagues from the University of Queensland, Australia, in 1999. It is currently implemented in over 25 countries and has been translated into over nine languages [87]. It is a very adaptable program, developed for a variety of ages from infant to teenagers. The risk level of the participants can vary as well, be it universal, selective, or indicated prevention or early intervention and treatment [111]. The unique aspect of the program is the fact that there are five levels of the program from community parent education meetings and parenting media to intensive individual in-home family sessions. Also, there is no set curriculum, just fact sheets that can be presented in any order: thus, helping the clinician to tailor the program to each family's individual needs. Clinicians work from these materials to individualize the family sessions [112] (Sanders et al. 2008). This makes it hard to evaluate the effect of dosage on outcomes. Triple-P Level One of community education meetings has been found on a CDC grant to reduce child maltreatment in a randomized 18 county sample in South Carolina [113]. The more intensive levels of Triple P are being evaluated by Dr. Prinz for child maltreatment prevention.

*(c) Early and Late Adolescence: Family Therapy or Family Skills Training Approaches.* These family therapy approaches include a number of individual family therapy programs mentioned earlier. They are flexible in length depending on the needs of the family. Family skills training programs have also been used successfully with preteens and adolescents, such as the SFP 10–14 Years and SFP 12–16 Years programs described below under the *Strengthening Families Program* section.

*Family Principle No. 3: Family Programs Are Most Enduring in Effectiveness If They Produce Changes in the Ongoing Family Dynamics and Environment.* Long-term positive outcomes are possible if families are willing to do their home practice lessons regularly, monitor their own behaviors and communication patterns, and have weekly family meetings to review weekly schedules, family business, chores, and plan fun family time together.

*Family Principle No. 4: Components of Effective Parent and Family Programs Include Addressing Family Relations to Increase Bonding and Attachment, Respectful and Clear Communication, and Parental Monitoring.* Virtually, all family EBPs focus on these critical content elements as also specified in the components of EBP above [88].

*Family Principle No. 5: Recruitment and Retention.* High rates are possible with families (80% to 85%) if the following are provided: (1) incentives for attendance, (2) a nonthreatening environment, (3) sensitive, well-trained and caring professional staff, and (4) culturally adapted program to match culture of the families [16]. Recruitment rates will vary by the type of program offered (longer program generally more difficult to recruit families), if recruiting agency has good relations or already provides services to families, types of recruitment methods used (direct contact best compared to letters or posters), time of day or week offered, and if clients can be mandated to attend by judges.

*Family Principle No. 6: Visual Materials Increase Learning and Family Interest.* Many of the best family EBPs have videos, CDs, or DVDs or web-versions that allow family member to see families demonstrating effective and ineffective ways to interact. For visual learners, this enhances learning and retention. Participants like racially matched families with up-to-date clothing and language. Computer interactive self-paced and self-testing CD, DVD, and web-delivered family programs demonstrate this principle such as *Parenting Wisely* developed for teens involved with the courts by Don Gordon [114]. The *Home Use SFP 7–17 Years DVD* video version has actually gotten better results with teens than the family group versions [115] possibly because teens are visual learners and not aggregated with other high risk youth together in groups to get a negative contagion effect.

*Family Principle No. 7: Competent Professional Staff with Parenting and Family Experience Are Best.* Agency directors have asked who would be the best people to select to staff family interventions. In our experience, program implementer or parent trainers who have backgrounds in family or group work and have been well trained in the model program do better in getting good results. They also need good clinical supervisors to meet with them at least weekly to review the progress of the program. It helps if staff shares the same general philosophy and background of the program. For therapy programs the personal characteristics of being personal, caring, and empathetic are critical. Our research [116] found with SFP that the best results are a balance of these warm and welcoming characteristics and also “work'em hard” characteristics that include being on time with good preparation, high expectations for family change and expecting them to do home practice lessons and family meetings. If there are two “warm and fuzzy” children's group leaders, the children's behaviors can actually get worse.

*Principles Are Not Enough to Prove Effectiveness*. Kumpfer and associates [15] stress the fact that programmes based on “principles of effective prevention,” while useful in designing new programmes, do not prove that a programme works. Proof is reserved for programmes tested in multiple randomized control trials and field trials with different populations and with different researchers and having large effect sizes for risk and protective factors or for the ultimate outcome desired.

### 4.3. Strengthening Families Program

The following description of *Strengthening Families Program (SPF)* developed by Dr. Karol Kumpfer at the University of Utah is used to illustrate how these principles of effective family program can result in a highly effective program. SFP is a highly structured family skills training program that is traditionally conducted in a 7- to 14-week multifamily group format involving three to four gender-balanced and culturally sensitive group leaders and a coordinator. Created and tested in the early 1980s on an NIDA research grant to help 6- to 11-year olds of substance abusers, later cultural and age adaptations have been developed. Recently, a new 10-session SFP 7–17 Years Home Use DVD and group versions have been developed and found amazingly effective with increased positive outcomes for adolescents compared to the regular 14-session SFP 12 to 16 Years lacking the DVD videos [115]. These DVD can be used very flexibly by individual clinicians or case workers to show 20-minute lessons on computers or TV that match the family's needs in clinics or their homes, or for family home use, or used in family group sessions.

SFP's effectiveness is attributed to the fact that the whole family attends each week thus changing the total family system. In the first hour, the children and parents attend their own classes with two gender-balanced group leaders. Children are trained in social and emotion-regulation skills, peer resistance skills, problem solving, effective communication, while parents receive training in “attention and rewards, clear communication, effective discipline, substance use education, problem solving, and limit setting [101] (Kumpfer, 2002).” Both learn about the importance of family play and togetherness time, effective family communications, and family meetings to enhance organization and reduce stress and conflict which practiced together in the second hour. Recruitment and retention are enhanced by removing many possible barriers to attendance by offering transportation, dinner, babysitting, and incentives for homework completion and graduation parties. Cultural adaptations by culturally sensitive group leaders are a mandated element of fidelity. At the very least, surface structure content should be altered to suit local cultural mores (i.e., incorporating songs, music, foods, games, and stories that reflect local mythology), including efforts to maximize community engagement by enhancing recruitment and retention [17]. The program has been tested with excellent results in a wide variety of community settings and schools [116], (Kumpfer, Alvarado, Whiteside, & Tait, 2005), youth and family centers, churches, public housing complexes, reservations, child welfare agencies [58], mental health centers, drug treatment agencies, probation services [15], juvenile courts, jails, prisons, hospitals, and recently refugee communities and shown to be appropriate for racial minority groups. Additional studies have examined barriers to implementation and offering a slate of remedies to attract hard-to-reach parents as well as minimize factors that impede adoption and program fidelity [117].

*Etiological Theory and Mechanisms of Effectiveness*. The theoretical rationale of SFP integrates family systems theory, social cognitive, and self-efficacy theory [118] with the developer's resilience framework model stressing importance of dreams, goals, and purpose in life in protecting youth from adversity [41, 117]. SFP improves the most salient risk and protective factors for substance use guided by its underlying etiological theory, the *Social Ecology Model of Adolescent Substance Abuse* [6]. This SEM-tested causal model found that the family cluster variables of family attachment or bonding, parenting skills and supervision, and communication of positive family values were the most critical in protecting youth from substance abuse and other negative developmental outcomes. While statistically significant for both boys and girls, the beta weights or impact was higher for girls [34].

*Effectiveness Studies of SFP*. The SFP has a long history of research demonstrating its effectiveness with numerous ages of children and ethnic populations. While originally developed and tested from 1982 to 1986 on a NIDA 4-group randomized component condition control trial (RCT) with 288 families as a *selective *prevention program for 6- to 12-year-old children of substance-abusers, the *universal* prevention school- and community-based versions have been found in eight RCTs by different research teams to be effective in reducing multiple risk factors and preventing adolescent alcohol and drug use [119–122]. Longitudinal follow-ups studies found 50% reductions in substance abuse, delinquency, depression, anxiety, and HIV risk even in genetically at risk youth years later in randomized school students whose families completed an African American cultural adaptation of SFP 10–14 Years, called Strong African American Families (SAAF) by Brody and associates [51–54]. Genetic risk was determined by saliva tests to identify the 40% of youth with one or two short alleles of the 5-HTTLPR serotonin transporter gene or the 7-repeat dopamine gene.

An independent quasi-experimental study of SFP in Utah with 800 families and 5- and 10-year follow-ups found long lasting positive improvements in families [123]. Considerable Type 2 translational effectiveness research in many states and counties finds SFP to be very robust when implemented widely. A statewide dissemination study with 1600 high risk families in New Jersey of the four age versions of SFP (3–5, 6–11, 10–14, and 12–16 Years) in 75 different community agencies found SFP 6–11 produced greatest effect sizes. The authors were surprised not to find a watering down of effect sizes compared to clinical RCT outcomes and commented that possibly using seasoned clinicians and prevention specialists who are experienced with their type of families, training, and online and phone supervision by program developers can produce larger behavior changes than typically found in RCTs often implemented by graduate student interns [124]. An RCT of SFP 6–11 Years in two Utah school districts compared to an 88-session teacher delivered skills training program (I Can Problem Solve) and found that those schools randomly assigned to get both programs had the best outcomes with almost additive effect sizes of each of the individual programs. SFP 6–11 Years outcomes did improve over the five-year time follow-up while the results for the youth only program decreases over time [119].

Replications of SFP in randomized control trials (RCTs) and quasi-experimental studies in different countries (United States, Canada, Ireland, UK, Netherlands, Germany, Greece, Spain, Portugal, France, Italy, Slovenia, Austria, Thailand, and Mexico) with different cultural groups by independent evaluators have found SFP to be an effective program in reducing multiple risk factors for later alcohol and drug abuse, mental health problems, and delinquency by increasing family strengths, children's social competencies, and improving parent's parenting skills [16]. For summary of outcomes from foreign studies see [17].

A review of school-based alcohol prevention by the Cochrane Systematic Review [12] concluded that SFP 10–14 Years is twice as effective in preventing alcohol misuse as any program with at least two years of follow-up data. These positive SFP outcomes are based on eight independent replications in NIAAA/NIDA/NIMH/CSAP-funded RCT with up to 10 years of follow-ups [119, 121]. Spoth and associates (2006) [122] report that not a single 6th grader getting SFP in Iowa reported meth use 10 years later compared to 3.2% in no-treatment youth. Multiple age and cultural adaptations of the SFP have found it to be robust in producing positive outcomes when culturally adapted for new populations [17]. SFP in now translated into multiple languages with cultural adaptations for 27 countries. Five quasi-experimental 5-year phase-in studies with African Americans, Hispanics, Pacific Islanders, and American Indians found that the cultural adapted versions had 40% better recruitment and retention because family values were respected. The UN Office of Drugs and Crime (UNODC) is disseminating SFP to developing countries in three regions of the world (Balkans, Central American, and Southeast Asia) and the Pan American Health Organization (PAHO) to Latin America. An RCT study funded by the Thailand government found that if both mothers and fathers attend versus only mothers, the outcomes are improved significantly.

A multicounty quasi-experimental evaluation of an Irish cultural adaptation for *indicated *prevention youth in Ireland [15] has produced some of the largest effect sizes (*d*. = .50 to 1.11). These Cohen's *d* effect sizes were about 20% larger than the comparison group US SFP norms possibly because the families were so needy and alcohol use rates higher at pre-test. All 21 measured teen, parent, and family outcomes were significantly improved in the 288 families. They used a unique collaborative coalition recruitment and staffing model that included probation services, local drugs task forces, schools, garda (police), and substance abuse treatment agencies. Each agency contributing a group leader was allowed to reserve 2 to 3 slots for families on their waiting lists for family services.

When not culturally adapted and implemented in high crime and disorganized communities, one RCT with 715 primarily high risk African Americans in the Washington, D.C. region, found reduced effect sizes and recruitment and retention [120]. Modifications to the program format and length are also considered to dramatically reduce fidelity. An unfortunate Swedish modification of the recommended 7-session SFP 10–14 Years format resulted in nonsignificant outcomes [125]. To save money after making expensive new videos, they eliminated all but two of the weekly family sessions and some of the parenting classes at night. Meals, incentives for homework completion, and babysitting were also not offered reducing parent attendance to only 33% of the families. The school teachers offer a longer SFP with more drug education classes to their regular full classroom of students; thus, increasing the possibility of a “negative contagion effect” described earlier by Dishion in his *Adolescent Transitions Program* when implementers are not skillful in maintaining control over the youth. This natural experiment thus demonstrated that a critical core component of SFP is improving the family relations by eating together, learning the same family strengthening skills, and playing games and role plays together to practice their interaction skills. Hence, the family skills training component is the “magically ingredient” in obtaining positive and lasting outcomes.

Cost-benefit studies [14, 126] report a positive cost/benefit ratio of $9.60 to $11 for SFP which underestimated the total benefit to the family because they were based only on benefits to just the student. However, by using a more efficient delivery system to effectively engage more families, the high cost of SFP at $1,000 per family can be considerably reduced to $4 per family for the DVD and handbook and about $100 if adding family coach.

Of interest also is the fact that Miller and Hendrie [14] also reported that no other substance abuse prevention program prevented as many adolescents from using substances. Their tables in the Appendix show that 18% of all youth participating in SFP will reduce or never initiate alcohol use compared to no-treatment youth and 15% for marijuana, 11% for other drugs, and even 7% tobacco. The next best prevention program was also a family program called *Adolescent Transitions Program* that prevented 14% of youth from using alcohol and 12% from using tobacco. These percentages of youth prevented from using alcohol and drugs were higher than all other family-focused or youth-only prevention programs. For example, *Life Skills Training* (LST) prevented 1% of youth from using alcohol and 3% from using marijuana. The *All Stars *program was the most cost beneficial youth only program at $32 saved per dollar spent. It prevented 11% of youth from using alcohol and 6% from using tobacco or marijuana. Spoth and associates [127] (2005) reported that at 22 years of age, lifetime diagnosed mental health problems (depression, anxiety, social phobias, and personality disorders) were reduced by 230% to 300% even 10 years after participation in SFP.

Recently SFP 6–11 Years was tested in a 5-year child maltreatment prevention study in Kansas with substance abusing parents. Researchers at the University of Kansas [58] reported in a propensity analysis that SFP reduced the days to family reunification dramatically from 258 days to 125 days, thus saving considerable foster care funds. The cost/benefit of this result is high.

### 4.4. Recruitment and Retention Issues and Solutions

Despite the increased effectiveness of family-based interventions, they can be difficult and expensive to implement on a large scale [128] particularly if home visits are involved. Most of the family interventions discussed in this chapter were originally based on individual family therapy techniques perfected in university clinics and then adapted as group-based prevention programs to reduce cost. Hence, clinicians needed to learn how to recruit lower risk families and to manage family group dynamics.

Recruitment is easier when implemented in clinics with high-risk parents concerned about their children. Also clinics typically have waiting lists. Community recruitment and retention of lower risk families are challenging until the implementers figure out “what works” for a particular population. This generally is a combination of ways to remove barriers and increase incentives for sign-up and attendance. Even hard-to-reach families will participate if they are provided transportation, baby sitting for younger and older children not in the program, meals or snacks, homework completions lotteries, weekly or small gifts for parents and children to attend, graduation gifts and a large graduation party, and money or coupons for research tests completion [117].

Also, the location and recruitment materials and messages have to be nonstigmatizing and the program activities and implementers engaging and fun for the whole family. Seeing improvements in family relations and children's behaviors and mental health is the major incentive for continuing in an effective family program.

If considered from a clinical treatment perspective, these family interventions have increased the reach of effective family therapy and skills training to more families, but when viewed from the perspective of *universal *prevention or public health environmental approaches, they do not impact as many students. When targeting all students in a classroom, these programs suffer from low attendance and can be perceived as stigmatizing [129]. In the NIDA longitudinal RCT comparing ISFP to PDFY in 6th grades in schools in southern Iowa, the average enrollment rate was 21.2% [130] and the rate of attendance in ISFP was only 16.9% of families. In the best circumstance, 38.5% of parents attended; in the worst circumstance, only 7.1% attended.

*Universal *family-based prevention programs have lower recruitment rates because many parents may not feel compelled to participate because there are no family problems driving them to enroll. Even if not, parents are increasingly just too busy to attend because of balancing hectic work schedules and family obligations.

Recruitment can be difficult in even *selective and indicated* prevention for higher risk families as well, but for different reasons. In *selective* prevention programs in high-risk environments, poverty, unemployment, low education, and socioeconomic disadvantage all factor into whether parents will participate [130].

In *indicated *family-based prevention, high risk prospective participants might feel reluctant to engage family or social service agencies or seek counseling support to remedy family problems for lack of trust, fear of losing their children, or resistance to change. Calling family interventions, “parenting or family classes” rather than therapy seem to increase interest in attending particularly if the families are mandated by the courts or strongly encouraged to attend by their case worker, therapist, or school counselors. For immigrant or ethnic families the lack of family services in their language or cultural sensitive recruitment methods and family services is a major deterrent to participation. The easiest families to recruit are those which are mandated by courts to attend a parenting class; however, these indicated parents will often select from a list the shortest and least effective class. Hence, the courts or CPS workers should only include the best family interventions in their lists of acceptable programs to attend.

Recruitment messages and methods also have to be tailored to the target populations. Most families respond best to personal recruitment methods from culturally matched recruiters or respected individuals that include letters, phone calls, face-to-face meetings, and emails. Community advertisements or posters can be low cost but will net fewer families.

All of these recruitment techniques to make the program more desirable and sensitive to family needs, reduce barriers to attendance, and also increase retention. The experience of evidence-based family program implementers is the fact that any family that attends the third session is likely to graduate unless they are overwhelmed by family issues. Retention also increases with increased experience delivering the program and good word-of-mouth in the community. Research on SFP even among difficult inner city Detroit heroin clients in multiple agencies and churches demonstrated that retention could increase to an average over 86% by the third or fourth SFP delivery from a low of about 40% the first time [131].

SFP and Fast Track also utilize snowballing techniques to attract new participants, inviting them to graduation ceremonies or recruitment meetings in an effort to spark further interest in other families. Other programs like Family Check Up (FCU) go to great lengths to train intervention staff using motivational techniques that encourage family participation and increase attraction to the program [132]. Avoiding negative labeling of participants as “high risk” or any reference to treatment should be stressed in training recruiters. As part of the advertising and promotion strategy, most EBP family interventions are billed as a positive resilience-building effort geared toward prosocial behavior and academic achievement, which enticed parents to participate and removed the stigma associated with “risk.” Family-centered programs involve a strong emphasis on parent and child empowerment and are vested in imbuing participants with feelings of increased self-efficacy, family harmony, and resilience.

### 4.5. Need for Cost-Effective Dissemination

One critical barrier to progress in field of prevention science is creating efficient dissemination systems [133] particularly for the more costly family evidence-based programs (EBP) in order to have a significant public health impact. Currently, most family interventions are offered in clinical or school settings by trained professionals that make them more expensive and limiting dissemination widely. Decreases in federal and state prevention funds have required creative solutions to increasing the dissemination of EBPs at reduced cost. Because of the advances in computer technology, some family program developers have begun creating lower cost CD, DVD, or web-versions of their programs. The intent is to scale-up their translation of EBPs from research to practice to achieve a broader public health approach [134, 135] and to improve their cost effectiveness. However, convincing low risk parents to participate in universal family versions will still be challenging as they see no need for a parenting class.

Another approach recommended by Santucci and associates [136] is direct marketing of EBPs to consumers as is done by pharmaceutical companies to create a demand for the adoption of EBPs by local community agencies. Of course consumers need accurate unbiased information or increased health literacy to make correct decisions about what to adopt for their situation since “one size does not fit all.” Vetting EBPs into website lists really helps as is currently available through federal, state, and national and international agencies, like the UNODC, Cochrane Reviews, WHO, and SAMHSA NREPP website lists. Still these web sites need to become more consumer-friendly like *Consumer Reports,* because many are difficult for even well-trained practitioners to digest to make accurate decisions on which EBP family interventions to adopt to match the needs of most of their families. Also few sites report clinical significance or effect sizes versus just statistical significance, so it is hard to determine which programs are the most effective. Search functions help that also include program length, time requirements, cost, and location of program delivery. “Channel partners” to help with marketing have been used by some family-based prevention programs, such as insurance companies, TV and radio, national organizations targeting alcohol, and drug prevention in teens such as the dissemination of the *Strengthening Families Program* by Mothers Against Drunk Driving (MADD), *Celebrating Families* (CF) by the National Association of Children of Alcoholics, and media partners like TV and radio stations for Triple P in Australia [110] (Sanders & Kirby, 2012).

The most promising approach to dramatically reduce cost and increase direct consumer marketing is the use of the Internet and computer technologies. DVD and web-based versions are being tested by several family-based prevention program developers. If effective, this computerized approach, has many advantages over group or individually delivered EBPs, including ease of availability for families to learn and review skills, standardized delivery with control of fidelity and quality, and dramatically reduced costs. A typical cost for a group delivered family intervention is about $1,000 to $2,000 per session for 7 to 20 sessions for 10 families or $100 per family per session. The new SFP 8–16 Year DVD costs only $4 per DVD for bulk orders including the color printed DVD *Guidebooks*. Of course, some other minor costs will be incurred in implementation such as funds for incentive lottery items for completion of weekly home practice lessons, graduation, and online evaluations.

Disadvantages of home-based DVD delivery are the loss of group and implementer support for behavior changes and social support for attendance and completion. However, this loss could be mitigated by social support methods such as families choosing “buddy-families” from among their children's friends to view the DVD lessons together or to chat/blog on dedicated SFP web site or Facebook pages. Regular family coach communication by cell phone, email, tweets, and Facebook will increase support for positive change. Not having high risk or difficult teens in a social skills group could have advantages in reducing negative contagion effects found by Dishion and associates for *Adolescent Transitions Program* (ATP). This lack of grouping high risk teens together could be one reason why the youth results were better for the Home Use SFP 8–16 Years DVD compared to a school-based group version of the same program in pilot studies by Kumpfer and Brown [115] (2012).

*Outcome Studies of PC-Based Delivery of Alcohol and Drug EBPs.*  While there are a few new family-based substance abuse EBPs being delivered through the use of computer technologies [114, 137–140] (Gordon, 2000; Haggerty, et al., 2007; Marsch, et al., 2007; Schinke, et al., 2004, 2009 a b c, in press), we could find only one study that compared group or individual clinical delivery for engagement and effectiveness with a PC-based version of EBPs, the Haggerty *Parents Who Care* parenting program. Only *Parenting Wisely* is being delivered through the web commercially, but we could find no outcome or feasibility studies for this program. Parenting Wisely is a family therapy intervention developed by Dr. Don Gordon [114, 141] (Gordon, 2000; Lagges, & Gordon,1999) as a spin off of Alexander's Functional Family Therapy clinical intervention for use with court-ordered adolescents. Dr. Steven Schinke's program used in Boys and Girls Clubs has been adapted for computer delivery on a CD as well [142] for HIV and substance abuse prevention. He has also developed a gender-specific family version for testing with inner city girls and their mothers in New York City with good results [140].

The outcome results from the 7-session *Parents Who Care (PWC) *bode very well for the effectiveness of computerized family interventions. PWC was tested as a self-paced video version with weekly phone calls with inner city African Americans and European Americans. In an intent-to-treat RCT design, it was compared to the group-based versions and no-treatment conditions [129]. PWC is an adaptation by Pollack and Haggerty on a NIDA SBIR grant of the prior reviewed Hawkins and Catalano *Preparing for the Drug-free Years,* which was found to be the second best alcohol misuse prevention program [12]. The surprising findings were the fact that the self-paced video version was more effective than the group-based version for the lower income and lower education level African American mothers, but not for the European American mothers. One hypothesis for the differences is that a self-paced home version allowed parents to repeat lessons thereby increasing learning. Another hypothesis is that the home-based program increased enrollment and retention because the families did not have to take public transportation to the group sessions and were able to complete and review sessions on their own time schedule.

If the use of computer delivered family interventions prove effective in future clinical trial RCTs, scientific knowledge, technical capability, and clinical practice will be improved through the use of innovative DVD-based delivery mechanisms of the more costly, but effective family interventions.

*Chapter Summary: Current and Future Directions in Family-Based Prevention*. This chapter has reviewed the causal and intervention theories underlying the success of family-based prevention programs and showcased a few of the most successful programs out of the hundreds that have been developed. In truth, we know a great deal about the causes of drug use and delinquency and have family programs with sufficient evidence to show that we can lessen the tremendous burden that crime causes on society through developmentally sound, theoretically consonant, and well-implemented family interventions. This chapter, then, is really a testament to the public health significance of family-centered prevention, perhaps the most cost-effective stopgap we have.

Because no one EBP family intervention is tailored to all family needs and to serve a broader range of youth ages and family risks, program developers have developed adapted universal, selective and indicated prevention program versions for different types of family pathology, risks, cultures, and ages. These efforts, which combine all levels of intervention, can do more, to relieve the burden of suffering that exists within socioeconomically disadvantaged families.

Needless to say there are advantages and disadvantages to all types of prevention programs regardless of target population and what intervention strategy is chosen. Notwithstanding, the evidence seems to indicate that family-based programs are successful when evaluated as efficacy trials in tightly controlled research settings and likewise when put into motion as effectiveness trials in real-world conditions. Although there are costs associated with any intervention, the real costs, the real burden to society is when we do not treat what is remediable.

## Figures and Tables

**Table 1:** Lifetime—16+ years Olds Use of Drugs. The five highest rates of use in each drug category appear in bold. Rates are reported as percentages.

Country	Cocaine	Cannabis	Tobacco	Alcohol
Colombia	**4.0**	10.8	48.1	**94.3**
Mexico	**4.0**	7.8	**60.2**	85.9
US	**16.2**	**42.4**	**73.6**	91.6
Belgium	1.5	10.4	49.0	91.1
France	1.5	**19.0**	48.3	91.3
Germany	1.9	17.5	51.9	**95.3**
Italy	1.0	6.6	48.0	73.5
Netherlands	1.9	**19.8**	**58.0**	**93.3**
Spain	**4.1**	**15.9**	53.1	86.4
Ukraine	0.1	6.4	**60.6**	**97.0**
Israel	0.9	11.5	47.9	58.3
Lebanon	0.7	4.6	**67.4**	53.3
Nigeria	0.1	2.7	16.8	57.4
South Africa	0.7	8.4	31.9	40.6
Japan	0.3	1.5	48.6	89.1
People's Republic of China	0.0	0.3	53.1	65.4
New Zealand	**4.3**	**41.9**	51.3	**94.8**

**Table 2:** Gender-sensitive drug use predictors in adolescents.

Gender-sensitive drug use predictors	More relevant	
	Girls	Boys
Depression		*✓*
Conduct disorder	*✓*	
Cigarette use	*✓*	
Maternal alcoholism	*✓*	
Maternal drug abuse	*✓*	
Low parental attachment	*✓*	
Low parental monitoring	*✓*	
Low parental concern	*✓*	
Unstructured home environment	*✓*	
Dysfunctional family	*✓*	
Smoking during pregnancy	*✓*	
Aggressiveness in first grade		*✓*
Higher anxiety response	*✓*	
Peer difficulties		*✓*
Childhood sexual abuse	*✓*	
